# α-Gal A missense variants associated with Fabry disease can lead to ER stress and induction of the unfolded protein response

**DOI:** 10.1016/j.ymgmr.2022.100926

**Published:** 2022-10-31

**Authors:** Francesco Consolato, Maurizio De Fusco, Céline Schaeffer, Federico Pieruzzi, Francesco Scolari, Maurizio Gallieni, Chiara Lanzani, Sandro Feriozzi, Luca Rampoldi

**Affiliations:** a Molecular Genetics of Renal Disorders Unit, Division of Genetics and Cell Biology, IRCCS Ospedale San Raffaele, Milan, Italy; b Nephrology and Dialysis Unit, ASST-Monza, San Gerardo Hospital and School of Medicine and Surgery, University of Milano-Bicocca, Monza, Italy; c Division of Nephrology and Dialysis, Department of Medical and Surgical Specialties, Radiological Sciences, and Public Health, University of Brescia and ASST-Spedali Civili, Brescia, Italy; d Nephrology and Dialysis Unit, ASST Fatebenefratelli Sacco, Department of Clinical and Biomedical Sciences, Università di Milano, Milan, Italy; e Chair of Nephrology, Vita-Salute University San Raffaele and Genomics of Renal Diseases and Hypertension Unit, IRCCS Ospedale San Raffaele, Milan, Italy; f Belcolle Hospital, Nephrology and Dialysis Unit, Viterbo, Italy; g School of Medicine, Vita-Salute San Raffaele University, Milan, Italy

**Keywords:** Fabry disease, α-galactosidase A, Missense mutations, ER stress, Unfolded protein response

## Abstract

Anderson-Fabry Disease (FD) is an X-linked lysosomal disorder caused by mutations in *GLA,* the gene encoding the lysosomal hydrolase α-galactosidase A (α-Gal A), leading to accumulation of glycosphingolipids in the lysosomes. FD is a multisystemic disorder leading to progressive cardiovascular, cerebrovascular and kidney dysfunction. Phenotypes are divided in two main classes, classic or non-classic, depending on substrate accumulation, age at onset, disease manifestation, severity and progression. The more severe classical phenotype is generally associated with mutations leading to absent or strongly reduced α-Gal A activity, while mutations with higher residual activity generally lead to the non-classical one. Approximately 70% of the over 1,000 Fabry disease-associated mutations are missense mutations, some leading to endoplasmic reticulum (ER) retention of mutant protein. We hypothesized that such mutations could be associated, besides the well-known absence of α-Gal A function/activity, to a possible gain of function effect due to production of a misfolded protein. We hence expressed α-Gal A missense mutations in HEK293 *GLA*^*−/−*^ cells and investigated the localization of mutant protein and induction of ER stress and of the unfolded protein response (UPR). We selected a panel of 7 missense mutations, including mutants shown to have residual or no activity *in vitro*. Immunofluorescence analysis showed that mutants with residual activity have decreased lysosomal localization compared with wild type, and partial retention in the ER, while missense mutants with no residual activity are fully retained in the ER. UPR (ATF6 branch) was significantly induced by all but two mutants, with clear correlation with the extent of ER retention and the predicted mutation structural effect. These data identify a new molecular pathway, associated with gain of function effect, possibly involved in pathogenesis of FD.

## 1 Introduction

Anderson-Fabry disease (FD) is a X-linked lysosomal storage disorder caused by mutations in *GLA* coding for α-galactosidase A (α-Gal A) [1]. α-Gal A is a lysosomal hydrolase whose deficiency results in the progressive accumulation of lysosomal glycosphingolipids, particularly globotriaosylceramide (Gb3) and globotriaosylsphingosine (lyso-Gb3).

Phenotypes are divided in two main classes, classic or non-classic. The classic form is associated with absent or strongly reduced α-Gal A activity, significant substrate accumulation in several districts, early onset during childhood or adolescence, progression to multiorgan damage leading to major complications such as end-stage kidney disease, hypertrophic cardiomyopathy, and cerebrovascular events. The non-classic form is characterized by higher residual α-Gal A activity, varying levels of substrate accumulation, late onset of clinical manifestations, including cardiac disease, kidney failure or cerebrovascular disease.

More than 1,000 Fabry disease–associated mutations have been identified. Approximately 70% of them are missense mutations. Based on their position in α-Gal A 3D structure they can be classified in 3 main classes: 1) active site mutations; 2) buried mutations, localized in the core of the enzyme inducing misfolding and premature degradation of the protein; 3) mutations mapping on the protein surface with milder effect on the folding [2]. Structural effect has a clinical correlate since active site and buried mutations associate with a more severe phenotype [3].

To date, two therapies are available for Fabry disease [4]. Enzyme replacement therapy (ERT) is based on intravenous administration of recombinant α-galactosidase or β-galactosidase. Even though this treatment showed to be effective, specific limitations are related to limited tissue penetration, no crossing of the blood–brain barrier, and development of anti-drug antibodies (ADA) reducing the enzyme activity. The second treatment is based on the use of the iminosugar 1-deoxygalactonojirimycin (migalastat), an orally administered small molecule analogue of the terminal galactose of Gb-3. Migalastat can reversibly bind to α-Gal A active site and act as a pharmacological chaperone, facilitating folding of mutant protein and increasing its ability to pass the quality control system of the endoplasmic reticulum (ER) and traffic to lysosomes. On the bases of cell assays, an estimated 35–50% of α-Gal A mutations are amenable to migalastat therapy [5].

Fabry disease is regarded as a loss of function disease, but the presence of missense mutations affecting α-Gal A folding suggests a possible gain of function effect. Indeed, ER retention of misfolded protein can lead to ER stress and possibly activation of the unfolded protein response (UPR). The primary function of the UPR is to restore cellular protein homeostasis through activation of 3 different sensors (PERK, IRE1, and ATF6) inducing downstream pathways aimed at reducing protein synthesis and increasing ER-associated folding and degradation. Chronic induction of the UPR, as in the case of continuous production of a mutated protein, can lead to cell death and/or activation of inflammation, and it has been shown to play a pathogenic role in several conformational diseases [6]. Its role in FD has been poorly studied with controversial results. Previous work analyzed the expression level of some ER stress markers in peripheral blood mononuclear cells of FD patients and controls without observing clear differences [7]. On the contrary, Braunstein et al. demonstrated activation of UPR in fly models expressing two α-Gal A mutant isoforms (A156V and A285D) compared with wild type animals [8].

In this study, we aimed at addressing this open question by characterizing ER stress and UPR in newly generated cellular models expressing different missense α-Gal A mutants. Our work clearly demonstrates that α-Gal A mutants associated with protein misfolding lead to ER retention and activation of the UPR to an extent that is correlated with structural impact and inversely correlated with residual activity of the investigated mutations. These results demonstrate a gain of function effect of α-Gal A mutation and suggest ER stress as a novel pathogenic pathway in FD.

## 2 Results

### 2.1 Generation of cell lines expressing different α-Gal A variants

**Fig. 1:**
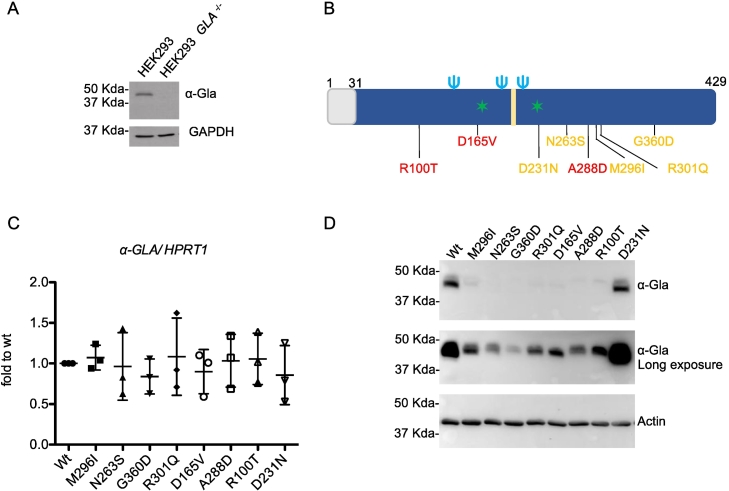
Expression of α-Gal A isoforms in transfected HEK293 *GLA*^*−/−*^cells. (A) Representative Western blot showing the absence of α-Gal A in Crispr/Cas9 edited cells. GAPDH was used as loading control. (B) Schematic representation of human α-Gal A. The grey box represents the leader sequence; the light blue trident represent glycosylation sites, represent active sites, the yellow box represents the substrate binding site. The position of selected variants is shown. In yellow, variants with residual activity; in red, variants with no residual activity. The residual activity of D231N variant is reported 0 or 0.5% of wild type activity [5,9]. (C) α-Gal A expression in transiently transfected HEK293 *GLA*^*−/−*^cells assessed by real-time RT-qPCR. Expression is normalized to *HPRT1*. Data are expressed as mean ± s.d. (*n* = 3 independent experiments). (D) Western blot analysis showing α-Gal A expression in lysates of HEK293 *GLA*^*−/−*^ cells transfected with different α-Gal A isoforms. Actin is shown as a loading control.

To model monoallelic expression of *GLA* we expressed wild type or missense α-Gal A isoforms in HEK293 cells that were depleted of *GLA* gene copies by Crispr/Cas9. In this model we were able to study the effect of α-Gal A variants without possible confounding effects due to the presence of the endogenous, wild-type protein. (Fig. 1A). These cells were transiently transfected with wild type or different α-Gal A mutant isoforms. We selected 7 missense mutations (M296I, N263S, G360D, R301Q, D165V, A288D and R100T) affecting the folding and stability of the protein. These mutations were selected on the bases of their residual activity as reported by previous studies [5]. We generated expression constructs for 2 mutants with residual activity about 15% of wild type protein (M296I, N263S), 2 mutants with residual activity lower or equal to 5% (G360D, R301Q) and 3 mutants with no residual activity (D165V, A288D and R100T). We also used a missense mutation affecting α-Gal A catalytic site (D231N) without significantly affecting protein stability, hence likely to be properly trafficked to the lysosome, but not functional [9] (Fig. 1B). This catalytic-dead mutant, with no enzymatic activity, was used as a control for any cellular phenotype associated with loss of α-Gal A function. Alpha Gal A expression was comparable in the different cells lines, as indicated by similar transcript levels measured by real-time RT-qPCR 72 h after transient transfection (Fig. 1C). When we analyzed protein levels we observed a clear difference between wild type and D231N mutant compared with all the other lines (Fig. 1D) that showed strongly reduced levels. This is in line with previous studies reporting rapid degradation of α-Gal A mutants affecting protein folding [[10], [11], [12], [13]].

### 2.2 α-Gal A misfolding mutations affect lysosomal localization to an extent that is proportional to their residual activity

**Fig. 2:**
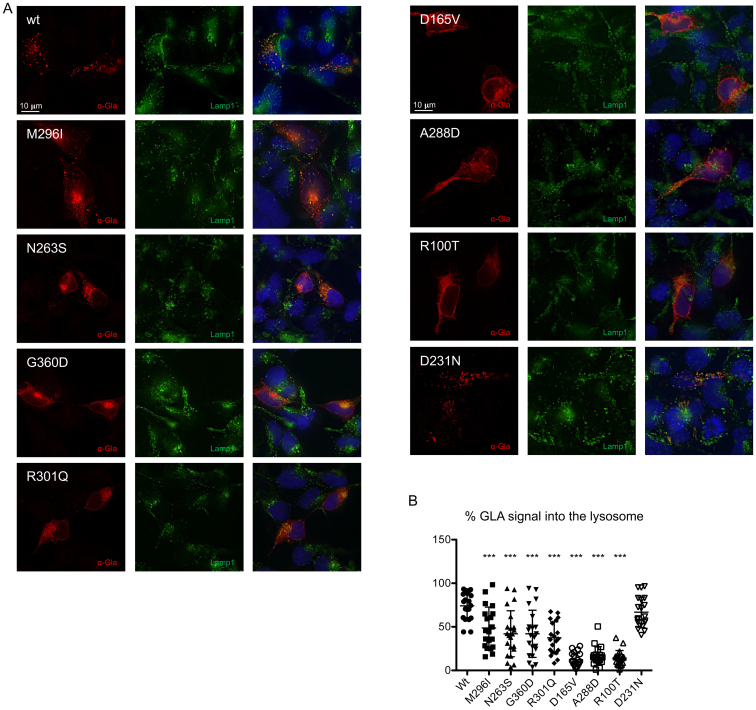
Intracellular localization of wild type and mutant α-Gal A isoforms. (A) Representative immunofluorescence images showing the cellular distribution of wild type or mutant α-Gal A (red) and of the lysosomal protein Lamp1 (green) in transiently transfected HEK293 *GLA*^*−/−*^ cells. (B) Quantification of α-Gal A (GLA) signal co-localizing with the lysosomal marker Lamp1 (*n* = 20 cells). Data are shown as vertical scatterplots indicating mean ± s.d. (all mutant isoforms were compared to wild type, one-way ANOVA followed by Dunnett's multiple comparison test). *** *p* < 0.001.

We investigated the relationship between the α-Gal A enzymatic activity and proper protein localization. Since α-Gal A is a glycosylated lysosomal protein, we quantified lysosomal localization of different α-Gal A isoforms through co-staining with the lysosomal marker Lamp1 (Fig. 2A). In cells expressing wild type and the catalytic mutant D231N, α-Gal A signal co-localized with Lamp1, demonstrating the expected lysosomal localization. On the contrary, for all missense mutants we observed significantly decreased levels of lysosomal α-Gal A. This effect was inversely correlated with the reported residual enzymatic activity of different mutants, i.e. mutants with minimal-to-low residual enzymatic activity showed partial lysosomal localization which was greatly reduced or virtually absent for mutants with no residual activity (Fig. 2B).

### 2.3 Missense α-Gal A variants are retained in the ER

**Fig. 3:**
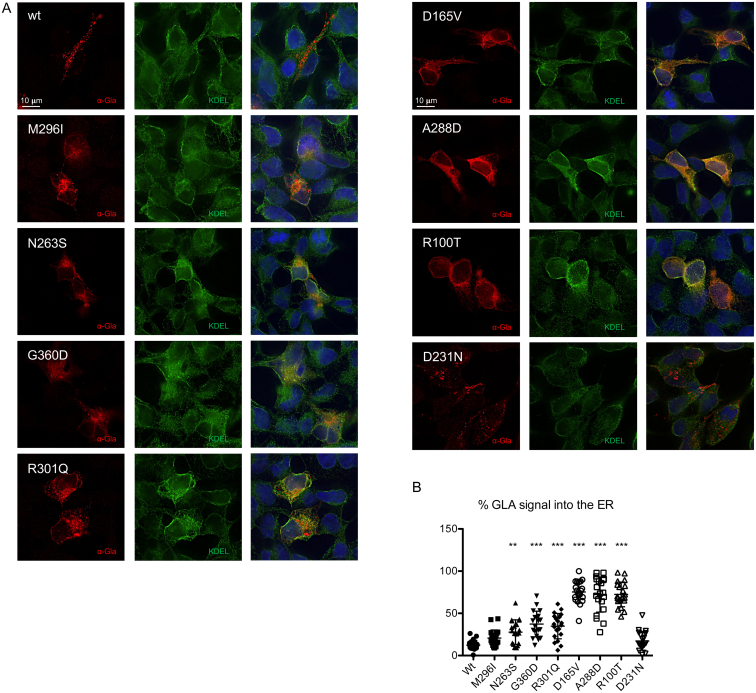
ER retention of missense α-Gal A mutants. (A) Representative immunofluorescence images showing the cellular distribution of wild type or mutant α-Gal A isoforms (red) and of the ER marker KDEL (green) in transiently transfected HEK293 *GLA*^*−/−*^ cells. (B) Quantification of α-Gal A (GLA) signal co-localizing with the ER marker KDEL (*n* = 20 cells). Data are shown as vertical scatterplots indicating mean ± s.d. (all mutant isoforms were compared to wild type, one-way ANOVA followed by Dunnett's multiple comparison test). **p < 0.01; *** *p* < 0.001.

To further investigate the subcellular localization of α-Gal A mutants we performed immunofluorescence analysis and quantified their co-localization with the ER marker KDEL (Fig. 3A). Missense mutants associated with residual enzymatic activity showed partial co-localization with the ER marker, supporting the possibility that reduced residual activity could be due to the amount of protein retained in the ER and hence not reaching the lysosome. In line with this idea, in cells expressing mutants with no residual activity we observed full co-localization of α-Gal A signal with KDEL, demonstrating that these mutants are fully retained in the ER (Fig. 3B).

### 2.4 Expression of α-Gal A missense mutants induces ER stress and the unfolded protein response

**Fig. 4:**
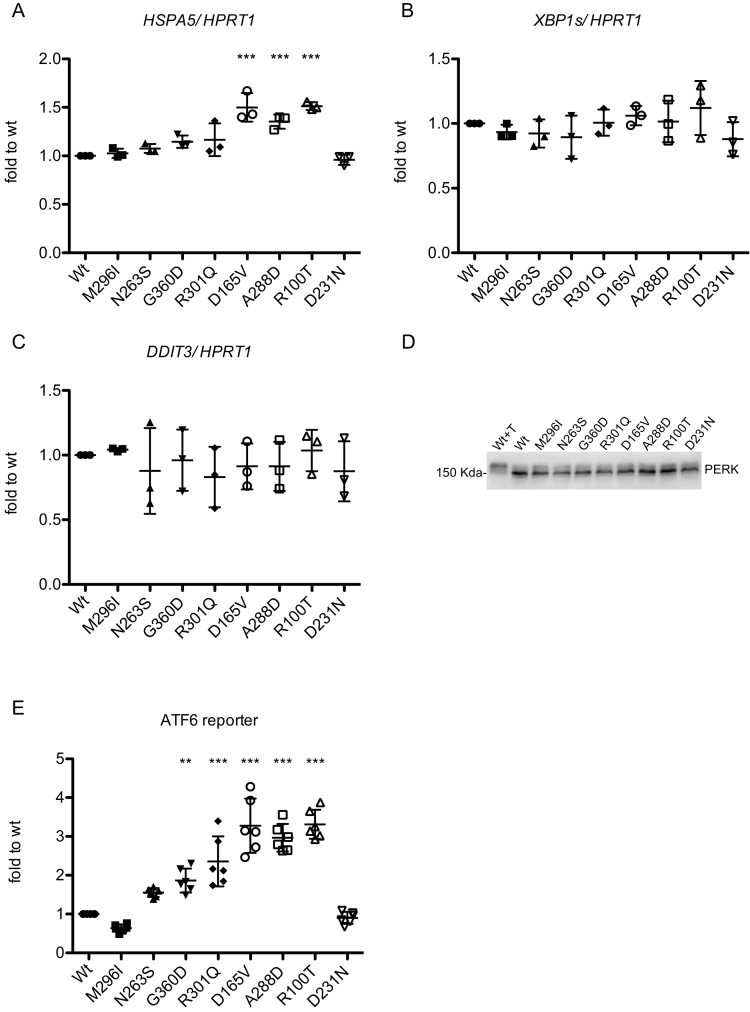
UPR induction in cells expressing missense α-Gal A mutations. (A-C) Real-time RT-qPCR showing expression levels of *HSPA5, XBP1s* and *DDIT3*, normalized to *HPRT1.* Data are expressed as mean ± s.d. (*n* = 3 independent experiments) relative to wild type cells. (D) Western blot analysis of PERK in HEK293 *GLA*^−/−^ cells transfected with different α-Gal A isoforms. T indicates incubation with tunicamycin (2 μg/ml for 14 h), as positive control of PERK phosphorylation (molecular weight shift). (E) ATF6 activation assessed through the use of a luciferase-based, ATF6 reporter construct. Data are expressed as mean ± s.d. (*n* = 6 independent experiments). (A-E) All mutant isoforms were compared to wild type, one-way ANOVA followed by Dunnett's multiple comparison test. **p < 0.01, ***p < 0.001.

To assess if ER retention of mutant variants induces ER stress and the UPR, we analyzed the expression level of *HSPA5* encoding the main ER chaperone Bip (Immunoglobulin heavy chain-binding protein). Real-time RT-qPCR shows a significant upregulation in all misfolded mutants with no residual activity, and a slight increase relative to wild type for one of the partially active mutants (R301Q), suggesting UPR induction in ER retained mutants (Fig. 4A). PERK and IRE1 branches of the UPR, assessed by analyzing the transcript level of *DDIT3* (CHOP) and spliced XBP1 (*XBP1s*) through real-time RT-qPCR and PERK phosphorylation by Western blot, are not induced in mutant-expressing cells compared with wild type ones (Fig. 4B-D). The activation of the ATF6 branch of the UPR was investigated by co-transfecting a well-established, luciferase-based construct [14]. This showed a robust induction of ATF6 activity in all non-active mutants, and a significant upregulation or a clear trend for upregulation in three out of four partially active mutants (N263S, G360D, R301Q) (Fig. 4E). Overall, these results show a significant induction of UPR (ATF6 branch) for all α-Gal A missense mutants that are fully retained in the ER, and for missense mutants that are partially retained in the ER, except for mutant M296I and for a clear trend for N263S.

### 2.5 Structural impact of α-Gal A mutations correlates with UPR induction

**Fig. 5:**
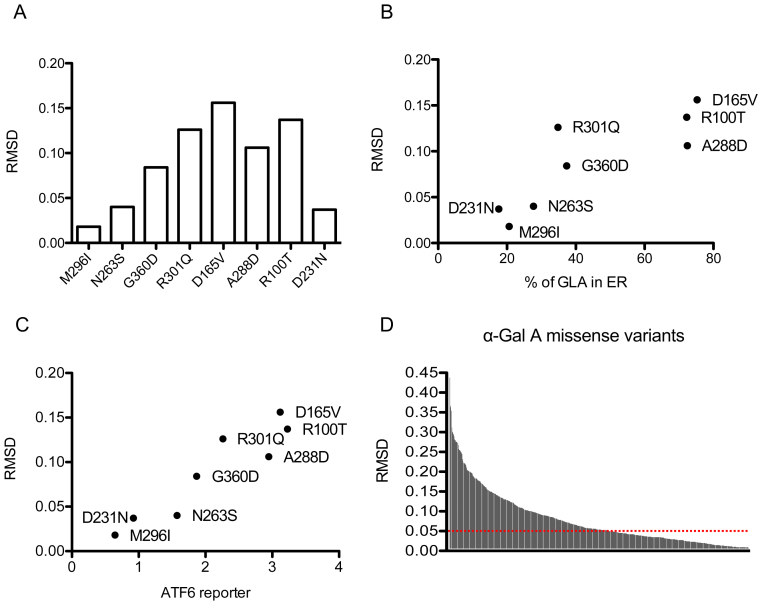
Predicted structural effect of Fabry disease missense mutations. (A) Histogram of root mean squared deviation (RMSD) values of α‑carbon atoms of the investigated missense mutants as a read out of their structural alteration, according to *fabry-database.org*. (B) Scatter plot of RMSD values and % of α-Gal A (GLA) in ER values of the selected missense mutations. (C) Scatter plot of RMSD values and ATF6 reporter values of the selected missense mutations. (D) Histogram of RMSD values of n = 663 missense mutations according to *fabry-database.org*. The horizontal dashed red line indicates 0.05 RMSD threshold.

Prompted by these findings we analyzed the predicted structural effect of selected mutations as reported in the Fabry mutation database (*fabry-database.org*). A previous study showed that structural features of mutations, in particular the root mean squared deviation (RMSD) of α‑carbon atoms relative to wild type protein [2,15], measuring the impact of mutations on protein structure, stability and catalytic activity, have a good prognostic value for clinical outcome [16]. RMSD values for the studied mutations clearly show a positive correlation with ER retention and ATF6 induction (correlation coefficient 0.85, *p* = 0.01; correlation coefficient 0.95, *p* = 0.001 respectively) (Fig. 5A-C). From this point of view, we could divide mutants into two groups: 1) mutants associated with clear UPR induction and high RMSD values (RMSD >0.05) (G360D, R301Q, A288D, R100T and D165V); 2) mutants with no significant ER stress induction and lower RMSD value (RMSD <0.05) (D231N, M296I and N263S).

Our data also suggest that a cutoff for RMSD, that could be set around 0.05 in our limited set of different variants, could separate UPR inducing from non-inducing mutations. By using this RMSD cut-off value we analyzed all missense mutations in the database (*n* = 663). Interestingly, about 50% (*n* = 324) of such mutations have a RMSD value above 0.05, hence may be associated with UPR induction (Fig. 5D).

## 3 Discussion

In this study we provide novel evidence demonstrating ER stress induction and UPR activation in a cellular model of Fabry-Anderson disease expressing α-Gal A missense variants with or without partial residual enzymatic activity.

We expressed wild type or missense α-Gal A isoforms in HEK293 cells that were knocked-out for endogenous *α-Gal A* gene to model monoallelic expression. In this system, despite comparable transcript level of all isoforms, we observed clearly reduced protein levels of mutants predicted to affect protein folding compared with wild type and the catalytic mutant D231N. These data are consistent with previously published studies showing degradation of unstable misfolded mutants.

Studying the localization of α-Gal A variants by immunofluorescence analysis with specific markers for the lysosome (LAMP1) or the endoplasmic reticulum (KDEL) we observed the expected lysosomal localization for wild type protein and the catalytic mutant D231N. On the contrary all missense mutants associated with residual activity showed decreased lysosomal localization and partial ER signal, while missense mutants without residual activity were essentially fully retained in the ER. These results are in line with a previous study on transiently transfected HeLa cells, showing that about 80% of D165V and A288D signal (fully ER retained in our study) and about 40% of M296I signal (partially retained in the ER in our study) localized in the ER [17]. Together, these results, demonstrate that residual/absent activity of α-Gal A misfolded mutants correlates with the extent of their trafficking impairment and ER retention.

The analysis of the expression of *HSPA5/BIP* showed upregulation in all misfolded mutants without residual activity, and a slight increase relative to wild type for one of the partially active mutants (R301Q), suggesting ER stress activation. The pivotal role of the ER chaperone BIP in response to ER stress is well established. Accumulation of misfolded proteins in the ER releases BIP from the ER transmembrane sensor proteins, PERK, IRE1, and ATF6, leading to the activation of UPR signaling cascade [18]. Consistently, we observed robust induction of the UPR, and of the ATF6 branch in particular, in all non-active mutants and a significant upregulation in two out of four partially active mutants (G360D, R301Q) compared with the wild-type isoform. ATF6 undergoes proteolytic cleavage that releases a cytosolic active form of the protein that migrates into the nucleus and acts as a transcription factor increasing transcription of genes related to the ER folding capacity, as ER chaperones (GRP94 and BIP) and disulphide isomerase PDI [19,20], and lipid metabolism [21].

To our knowledge, studies on UPR activation in Fabry disease are yet limited and with controversial findings [7,8]. Our study provides an extensive characterization of different α-Gal A missense mutants, selected for their reported residual activity, clearly supporting activation of UPR in FD. The fact that we observed activation of the ATF6 branch only, as opposed to activation of all UPR branches reported in the fly model, may be due to several factors, including the different mutations that were investigated or the different model systems. Nevertheless, activation of ATF6 only is not unprecedented, as it was described for instance for ER-retained nephrin mutants associated with congenital nephrotic syndrome of the Finnish type. The specific induction of the ATF6 branch was suggested to be an adaptive/cytoprotective mechanism to counteract ER stress [22]. Activation of specific UPR transcriptional signatures differing among tissues or organs and among human and mouse supports the importance of testing activation of these pathways in different systems [23]. It is hence possible that α-Gal A could activate other branches of the UPR if tested in different settings.

**Fig. 6:**
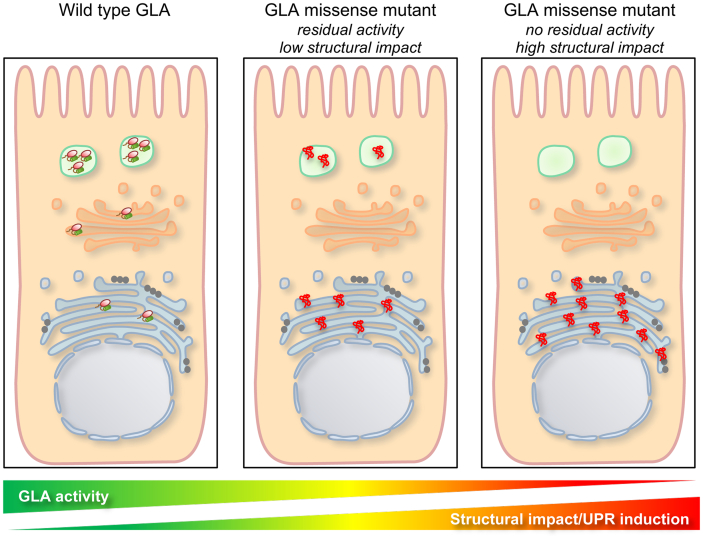
Proposed model of UPR induction, mutation structural impact and GLA residual activity. In wild type condition, α-Gal A enters the secretory pathway to reach its lysosomal localization. At steady state the protein is almost completely localized in the lysosome and active. Misfolded mutants reported to have residual activity and low structural impact are partially ER retained inducing ER stress and UPR to an extent that is inversely correlated with ER retention and residual enzymatic activity. Mutants shown to have high structural impact and no residual activity are fully retained in the ER and induce robust ER stress and UPR.

In our study two mutants, M296I and N263S, did not significantly induce the UPR, even though a trend for induction is evident for the N263S variant. Of note, M296I is a particularly mild mutation: it is associated with late-onset and mild clinical manifestation and the plasma Lyso-Gb3 level in patients is lower than in classic and other late-onset FD males, although higher than controls [24]. Hence it is conceivable that its structural effect, affecting M296 in the core region of α-Gal A, could be particularly mild. This is indeed observed when taking into account the impact of all investigated mutations on the structure of the protein by considering their root mean squared deviation (RMSD), a value indicating the structural distance between the variant and the wild type protein [2]. The M296I and N263S variants have lower RMSD compared with the other variants, similar to the one of the catalytic mutant D231N that is not predicted to affect protein structure. Interestingly RMSD values are correlated with ATF6 induction, supporting the link between missense variants structural effect, residual activity, extent of ER retention and UPR induction (Fig. 6). Along this line we collected available RMSD values for over 600 missense variants and, using a RMSD cut-off value of 0.05, we reveal that about 50% of them have a structural impact that could be associated with UPR induction. We acknowledge that this observation is preliminary and based on a relatively small set of mutations analyzed in this study. Additional studies, envisaging high-throughput analysis of UPR induction on large panels of mutants, are certainly warranted to understand the relevance of this finding.

UPR activation in a lysosomal disease due to ER retention of the mutated enzyme is not unprecedented. For instance, in Gaucher disease, due to mutations in the *GBA1* gene, encoding lysosomal acid β-glucocerebrosidase (GCase), the expression of mutant GCase in different systems, including patient fibroblasts and fly models, induces ER stress and activation of the UPR [25]. In the context of FD, additional studies will be required in order to establish the role of UPR induction, i.e. if it is an adaptive pathway to counteract mutant protein accumulation in the ER or if it has a maladaptive role contributing to disease pathogenesis. Several studies on different proteinopathies clearly demonstrated that a possible detrimental effect downstream of chronic UPR induction is inflammation that plays an important role in FD pathogenesis [26]. Pro-inflammatory pathways in FD have been assumed to be stimulated by unmetabolized glycolipid substrates. However, our data suggest that UPR could be envisaged as an additional candidate mechanism eliciting inflammation. Defining the role of UPR will be relevant for mechanistic dissection of disease pathogenesis and, since UPR branches can be specifically modulated, it may have translational relevance by providing a possible synergetic approach to existing therapies. Of note, among the mutations that were investigated in this study, four are amenable to treatment with the pharmacological chaperone migalastat (M296I, N263S, G360D, R301Q) while three are not amenable (D165V, A288D, R100T) [27]. Hence, UPR modulators could be envisaged in combination with pharmacological chaperone or ERT.

In sum, our study demonstrates that α-Gal A missense mutations can induce ER stress and the UPR suggesting that FD is not only a lysosomal storage disease due to lack to GLA activity and substrate accumulation, but it also has a gain of function component due to ER retention of mutant protein.

## 4 Methods

### 4.1 Cell lines and culture conditions

Human Embryonic Kidney 293 (HEK293) cells were grown in DMEM supplemented with 10% fetal bovine serum (Euroclone, Pero, Italy), 200 U/ml penicillin, 200 μg/ml streptomycin, and sodium pyruvate 1 mM at 37 °C with 5% CO_2_.

### 4.2 Generation of HEK293 *GLA*^−/−^ cells

HEK293 cells knock-out for the *GLA* (HEK293 *GLA*^*−/−*^ cells) were generated as follows. We used the Invitrogen TrueDesign Genome Editor to design guide RNA (Thermofisher, Waltham, MA, USA). Invitrogen ™ TrueGuide ™ Synthetic guide RNA (5’-ACCCTCAGCGCTTTCCTCAT-3′) and the TrueCut Cas9 Protein V2(A36496) were transfected in HEK293 cells using Lipofectamine™ CRISPRMAX™ Cas9 Transfection Reagent according to the manufacturer's protocol. After 48 h transfected cells were subcloned and clonal cell populations were then tested for α-Gal A expression by Western blot. A clone showing no α-Gal A signal was selected for further studies.

### 4.3 Constructs

GLA_OHu19239C_pcDNA3.1(+) -C-HA was purchased by GenScript (Piscataway, NJ, USA). C-terminal HA tag was abolished by introducing a stop-codon before the HA sequence through directed mutagenesis by QuikChange Lightning Site-Directed mutagenesis kit (Agilent Technologies, Santa Clara, CA, USA) according to the manufacturer's protocol using the following primers: Forward: 5’-CAATGCAGATGTCATTAAAAGACTTACTTTGACCCTACCCATACGATG-3′; Reverse: 5’-CATCGTATGGGTAGGGTCAAAGTAAGTCTTTTAATGACATCTGCATTG-3′. The construct was verified by Sanger sequencing.

The expression vector for untagged, wild-type α-Gal A was mutagenized to generate the selected mutants using QuikChange Lightning Site-Directed mutagenesis kit.

Primers for each mutation are listed below.

**Table:** 

	Sequence
M296I_Fwd	5'-CATGGCTGCTCCTTTATTCATATCTAATGACCTCCGAC-3'
M296I_Rev	5'-GTCGGAGGTCATTAGATATGAATAAAGGAGCAGCCATG-3'
N263S_Fwd	5'-TGGACCAGGGGGTTGGAGTGACCCAGATATG-3
N263S_Rev	5'-CATATCTGGGTCACTCCAACCCCCTGGTCCA-3'
G360D_Fwd	5'-CCGGCAGGAGATTGATGGACCTCGCTCTT-3'
G360D_Rev	5'-AAGAGCGAGGTCCATCAATCTCCTGCCGG-3'
R301Q_Fwd	5'-CATGTCTAATGACCTCCAACACATCAGCCCTCAAG-3'
R301Q_Rev	5'-CTTGAGGGCTGATGTGTTGGAGGTCATTAGACATG-3'
D165V_Fwd	5'-TTGCTGACTGGGGAGTAGTTCTGCTAAAATTTGATGG-3'
D165V_Rev	5'-CCATCAAATTTTAGCAGAACTACTCCCCAGTCAGCAA-3'
A288D_Fwd	5'-GATGGCCCTCTGGGATATCATGGCTGCTC-3'
A288D_Rev	5'-GAGCAGCCATGATATCCCAGAGGGCCATC-3'
R100T_Fwd	5'-GTTGGATGGCTCCCCAAACGGATTCAGAAGGCAGACTT-3'
R100T_Rev	5'-AAGTCTGCCTTCTGAATCCGTTTGGGGAGCCATCCAAC-3'
D231N_Fwd	5'-CAATCACTGGCGAAATTTTGCTAACATTGATGATTCCTGG-3'
D231N_Rev	5'-CCAGGAATCATCAATGTTAGCAAAATTTCGCCAGTGATTG-3'

All constructs were verified by Sanger sequencing.

### 4.4 Real-time RT-qPCR

HEK293 *GLA*^*−/−*^ cells were grown in 12-well plates in complete medium and transiently transfected using Lipofectamine 2000 (Life Technologies) according to the manufacturer's protocol. RNA was extracted 72 h after transfection by using PRImeZOL ™ reagent following the manufacturer's protocol. RNA was reverse-transcribed using iScript™ gDNA Clear cDNA Synthesis Kit (Bio-Rad, Hercules, CA, USA) following the manufacturer's protocol. Real-time RT-qPCR was performed on the CFX96 Touch Real-Time PCR Detection System (Bio-RAD) using the qPCR Core kit for SYBR® Green I No ROX (Eurogentec, Liège, Belgium) with specific primers for the indicated genes (*pGLA* refers to transfected *GLA*).

**Table:** 

	Forward	Reverse
*HSPA5*	5’-CGCTGAGGCTTATTTGGGAAA-3′	5’-TGCCGTAGGCTCGTTGATG −3’
*XBP1S*	5’-GAGTCCGCAGCAGGTG-3′	5’-ATACCGCCAGAATCCATGG-3’
*DDIT3*	5’-GCTGGAACCTGAGGAGAGAG-3′	5’-TTCTTCCTCTTCATTTCCAG-3′
*pGLA*	5’-ACAGCTCCTCCCTGTGAAAAG-3’	5’-GATTACGCTTGATAAACCCGCTG-3’
*HPRT1*	5’-AGCCCTGGCGTCGTGATTAGT-3’	5’-TGTGATGGCCTCCCATCTCC-3’

### 4.5 Western blot

HEK293 *GLA*^*−/−*^ cells were grown in 12-well plates in complete medium, transiently transfected as indicated above and lysed 72 h later using 100 μl of lysis buffer (20 mM Tris-HCl, pH 7.4, 50 mM NaCl, 1% Triton, 1 mM EDTA, 0.05% sodium dodecyl sulfate, 10 mM NaF, 0.5 mM sodium orthovanadate, 1 mM glycerophosphate and protease inhibitor cocktail) (Sigma-Aldrich, St Louis, MO, USA) for 1 h at 4 °C under rotation followed by centrifugation 10 min at 17,000*g*. Soluble fractions were quantified by the BioRad Protein Assay (Bio-Rad).

Twenty μg of each protein lysate were analyzed by reducing SDS-polyacrylamide gel electrophoresis (PAGE). Transblotted nitrocellulose membranes (GE Healthcare,Chicago, IL, USA) were incubated with the indicated primary antibody followed by incubation with horseradish peroxidase-conjugated secondary antibody (1:5000 dilution; GE Healthcare). Protein bands were visualized with the Immobilon Western Chemiluminescent Horseradish Peroxidase Substrate kit (Millipore, Billerica, MA, USA).

### 4.6 Immunofluorescence

HEK293 *GLA*^*−/−*^ cells were transiently transfected as indicated above, grown on coverslip in 24-well plate for 72 h and then fixed in 4% paraformaldehyde (PFA) for 30 min. Cells were permeabilized for 30 s with cold MetOH. After washing in PBS, cells were incubated 30 min at room temperature in 10% preimmune donkey serum-0.1% Triton X-100 in PBS. Cells were then incubated for 1 h at room temperature with the indicated primary antibodies. Cells were then washed in PBS and incubated for 1 h at room temperature with the appropriate secondary antibody: Alexa Fluor 594-conjugated donkey secondary antibody against rabbit immunoglobulin G (IgG) (dilution 1:500; Invitrogen); or Alexa Fluor 488-conjugated donkey secondary antibody against mouse IgG (dilution 1:500; Invitrogen). Cells were then stained for 5 min with 4,6-diamidino-2-phenylindole and mounted using FluorSave Reagent (Calbiochem, San Diego, CA, USA). All slides were acquired by GE healthcare DeltaVision™ Ultra microscope.

### 4.7 Image quantification

*Z*-stacks at 3 μm interval were acquired for each selected field at high magnification (60×) using DeltaVision Ultra microscope (GE healthcare). After acquisition each image was deconvoluted by SoftworX 7.2.0 software (GE healthcare).

Z-stack files were imported into Volocity® software (Quorum technologies, Puslinch, Canada) and for each isoform 20 GLA-expressing cells were analyzed to quantify the entire volume of GLA signal, the entire volume of lysosome marker LAMP1, or the entire volume of ER marker KDEL, and the volume of GLA signal present in lysosomes or ER, expressed as “% of total GLA signal into the lysosome (or ER)”. To calculate the volume of lysosomes, ER or GLA signal, specific regions of interest (ROI) were manually drawn around the cells and a semi-automated threshold was used to quantify positive voxels over background.

### 4.8 Antibodies

Rabbit monoclonal anti-galactosidase alpha antibody (ab 168341; Abcam, Cambrige, UK; for Wb 1:3000; for IF 1:500); mouse monoclonal anti-GAPDH antibody (sc-32233, Santa Cruz Biotechnology, Dallas, TX, USA; for Wb 1:1000;), anti-beta-actin-peroxidase conjugated (A3854, Merck, Burlington, MA, USA; for Wb 1:100,000), mouse anti-KDEL (10C3, Enzo Life Sciences, NY, USA; for IF 1:500); mouse anti-LAMP1 (H4A3: Developmental Studies Hybridoma Bank, Dshb, Iowa, IA, USA; for IF 1:500); rabbit monoclonal anti-PERK (#3192, Cell Signaling, Danvers, MA, USA for Wb 1:1000).

### 4.9 ATF6 activity

The reporter construct for ATF6, p5xATF6-GL3, was created by Prof. Ron Prywes [14] and was obtained from Addgene (Watertown, MA, USA) (Addgene plasmid # 11976). HEK293 *GLA*^*−/−*^ cells were plated in 24-well plate and transfected with 250 ng of vector for each α-Gal A isoform, 250 ng of ATF6 reporter construct and 5 ng of pGL4.73[hRluc/SV40] (Promega, Madison, WI, USA) using lipofectamine 2000 (Thermofisher). Firefly and Renilla luciferase were measured 72 h after transfection using the Dual-Luciferase® Reporter Assay System (Promega). ATF6 activity was expressed as Firefly signal normalized to Renilla signal.

## Declaration of Competing Interest

This work was supported by a grant from 10.13039/100015362Amicus Therapeutics, United States [Investigator Initiated Program] (to L.R.). The funding source had no involvement in the research here presented at any stage.

## Data Availability

Data will be made available on request.
